# Whole-genome landscape of histone H3K4me3 modification during sperm cell lineage development in tomato

**DOI:** 10.1186/s12870-024-05318-8

**Published:** 2024-06-27

**Authors:** Yunyun Song, Zhikai Chang, Yixuan Feng, Tai Wang, Lingtong Liu

**Affiliations:** 1 Key Laboratory of Plant Molecular Physiology, Institute of Botany, Chinese Academy of Sciences, Beijing, 100093 China; 2 College of Life Science, University of Chinese Academy of Sciences, Beijing, 100049 China; 3 China National Botanical Garden, Beijing, 100093 China

**Keywords:** H3K4me3, Pollen, Microgametogenesis, Histone modification, Tomato

## Abstract

**Background:**

During male gametogenesis of flowering plants, sperm cell lineage (microspores, generative cells, and sperm cells) differentiated from somatic cells and acquired different cell fates. Trimethylation of histone H3 on lysine 4 (H3K4me3) epigenetically contributes to this process, however, it remained unclear how H3K4me3 influences the gene expression in each cell type. Here, we conducted chromatin immunoprecipitation sequencing (ChIP-seq) to obtain a genome-wide landscape of H3K4me3 during sperm cell lineage development in tomato (*Solanum lycopersicum*).

**Results:**

We show that H3K4me3 peaks were mainly enriched in the promoter regions, and intergenic H3K4me3 peaks expanded as sperm cell lineage differentiated from somatic cells. H3K4me3 was generally positively associated with transcript abundance and served as a better indicator of gene expression in somatic and vegetative cells, compared to sperm cell lineage. H3K4me3 was mutually exclusive with DNA methylation at 3’ proximal of the transcription start sites. The microspore maintained the H3K4me3 features of somatic cells, while generative cells and sperm cells shared an almost identical H3K4me3 pattern which differed from that of the vegetative cell. After microspore division, significant loss of H3K4me3 in genes related to brassinosteroid and cytokinin signaling was observed in generative cells and vegetative cells, respectively.

**Conclusions:**

Our results suggest the asymmetric division of the microspore significantly reshapes the genome-wide distribution of H3K4me3. Selective loss of H3K4me3 in genes related to hormone signaling may contribute to functional differentiation of sperm cell lineage. This work provides new resource data for the epigenetic studies of gametogenesis in plants.

**Supplementary Information:**

The online version contains supplementary material available at 10.1186/s12870-024-05318-8.

## Introduction

In flowering plants, pollen (male gametophyte) development from the haploid microspore involves two rounds of mitosis. The asymmetric division of microspores is determinative, by which a microspore generates a larger vegetative cell (VC) and a smaller generative cell (GC) embedded in the VC, thus forming a unique cell-within-a-cell structure. The two types of cells have distinct identities and fate: the GC undergoes one more round of mitosis to produce two sperm cells (SCs), whereas the VC no longer divides and terminally differentiates into pollen tubes to deliver SCs into the embryo sac for double fertilization [1–3]. The series of male reproductive cell populations from microspores to GCs and finally to SCs represents a well-characterized SC lineage [4–6]. Growing evidence has shown that the development of plant and animal reproductive cells from surrounding somatic cells is closely associated with epigenetic regulation of histone modifications and DNA methylations [7–9].

During the male gametophyte development of flowering plants, a variety of histone modifications are linked to gene expression [10–12]. Histones are small positively charged proteins that package genomic DNA into nucleosomes, which wrap approximately 146 ~ 147 bp of DNA around an octamer of four core histones: H2A, H2B, H3, and H4 [13]. The core histones undergo diverse post-translational modifications: including methylation, acetylation, phosphorylation, ubiquitination, and sumoylation. These modifications can change the accessibility of DNA and the recruitment of various effector proteins related to transcription, thereby altering gene expression indirectly [14–16]. The majority of histone methylations are located on the histone tails which are intrinsically disordered and serve as signaling antennas of chromatin [14]. Histone methyltransferase (HMT) catalyzes the methylation of lysine and arginine residues of histones by using S-adenosyl methionine (SAM) as a major methyl donor. Up to three methyl groups can be transferred to the same lysine residues and several lysine residues on the same tail can be simultaneously methylated. The potential effects of lysine methylation on transcription depend on the position and degree of methylation. In general, H3K4, H3K36, and H3K79 methylations are linked to transcriptional activation, whereas H3K9, H3K27, and H4K20 methylations are associated with heterochromatinization and transcriptional silencing [17–20]. Certain histone methylations that correlate with transcript abundance can be used for the prediction of gene expression. In human CD4^+^ T-cells, H3K4me3 and H3K79me1 were most predictive for the expression of low CG content promoters [21], whereas H3K4me3 and H3K79me3 were the most predictive for non-CG-related promoters [22].

H3K4me3 is enriched in actively transcribed regions and is generally considered a conserved hallmark of active transcription [23–26]. Recent evidence suggests H3K4me3 plays a key role in facilitating the release of paused RNA Polymerase II and elongation rather than transcription initiation [27]. In animals, it has been shown that basal transcription factor TFIID could bind to the H3K4me3 mark and selective loss of H3K4me3 reduces transcription from a subset of promoters in vivo [28]. However, H3K4 methylation is dispensable for cell viability in *Saccharomyces cerevisiae* [29] and disruption of H3K4 methylation only caused limited changes in gene expression in *Drosophila* [30]. In plants, dynamic regulation of H3K4me3 is tightly associated with various developmental processes, such as seed dormancy and germination, root patterning, leaf senescence, flowering time control, gametogenesis, and response to stresses [31–35].

In Arabidopsis, SET domain-containing histone methyltransferases Arabidopsis Trithorax 1 (ATX1), SET DOMAIN GROUP2 (SDG2)/Arabidopsis Trithorax Related 3 (ATXR3) are required for H3K4me3 methylation [36]. ATX1 is required for the specific deposition of H3K4me3 marks at the *FLOWERING LOCUS C (FLC)* locus which controls the transition from vegetative to reproductive development [35]. During the somatic-to-reproductive cell fate transition, the H3K4me3 was enriched in the megaspore mother cell and likely contributed to the postmeiotic development of the female germline [9]. In Arabidopsis, the loss of SDG2 led to impaired pollen mitosis microgametogenesis. SDG2-mediated H3K4me3 is involved in the maintenance of the decondensed chromatin state of pollen VC, possibly by preventing ectopic heterochromatic H3K9me2 speckle formation [32]. In tomato, we have shown that SC lineage development is accompanied by progressive loss of the active marks H3K4me3 and accumulation of the repressive methylation mark H3K9 methylation [4]. However, it remained unclear how H3K4me3 modification influences the gene expression of the sperm cell lineage and the VC.

Here, based on our previously established procedure [37], we isolated microspores and nuclei of GCs, SCs, and VC at high purity and obtained epigenomic profiles of histone H3K4me3 modifications by using chromatin immunoprecipitation with sequencing (ChIP-Seq). This study was performed using *Solanum lycopersicum* (Heinz1706), a tomato cultivar with a reference genome sequence [38]. We found the distribution of H3K4me3 primarily enriched in the promoter regions, especially at immediate downstream of transcription start sites (TSS), which were highly concurrent with transcriptional activation. H3K4me3 is mutually exclusive with DNA methylation at 3’ proximal of the TSS, but not in other positions of the transcribed region. Unexpectedly, an increasing number of H3K4me3 peaks were detected during the development of SC lineage cells and the VC showed a remarkably lower level of H3K4me3 enrichment as compared with GCs and SCs. The microspore maintained the features of somatic tissue, while significant loss of H3K4me3 in genes related to brassinosteroid signaling and cytokinin signaling occurred in GCs and VC, respectively. Furthermore, our data demonstrated that H3K4me3 deposition is a better indicator of active gene expression in somatic cells and VC than in GCs and SCs.

## Materials and methods

### Plant materials

The tomato (*Solanum lycopersicum*) cultivar Heinz 1706 was planted in a greenhouse at 28℃ for four weeks, then fully expanded leaves were collected. The vegetative nuclei (VN), generative nuclei (GN), and sperm nuclei (SN) were isolated from germinated tomato pollen as described previously [37]. In brief, mature pollen grains from tomato at anthesis were harvested, pre-hydrated with saturated Na_2_HPO_4_, then incubated in germination medium (20 mM MES, 3 mM Ca(NO_3_)_2_, 1 mM KCl, 0.8 mM MgSO_4_, 1.6 mM H_3_BO_3_, 24% PEG 4,000, 2.5% sucrose, pH 6.0) at 25℃ in the dark with shaking at 90 rpm. Vegetative nuclei were isolated from 1.5-h cultured pollen tubes, and further washed with ice-cold isolation buffer (10 mM MES, 10 mM NaCl, 5 mM EDTANa_2_, 0.5 mM Spermidine, 0.15 mM Spermine, 1 mM DTT, 18% Sucrose, pH 6.0). Generative cells and sperm cells were isolated from just-germinated (20 min) pollen and 10-h cultured pollen tubes, respectively. The isolated GCs and SCs were resuspended in ice-cold isolation buffer supplemented with 0.1% Triton X-100, 10 mM β-mercaptoethanol and 1× complete protease inhibitor cocktail (Roche) at 4℃ for 10 min, then the suspension was centrifuged at 1,500 g for 5 min at 4℃ to collect GN and SN. For isolation of microspores, 5-mm long anthers were picked and sliced with a stirring bar to release microspores into 0.6 M mannitol. The microspore suspension was filtered through a 48-µm nylon mesh to remove debris, then centrifuged at 500 g for 2 min at 4 °C to collect spores. Isolated VN, GN, SN, and microspores were flash-frozen in liquid nitrogen and stored at -80℃.

### Immunofluorescence

Briefly, SC lineage samples were fixed in 1×phosphate-buffered saline (PBS) with 4% paraformaldehyde at 4 °C for 2 h. After fixation, MS, GCs, and SCs were washed once with PBS, dropped onto poly-L-lysine slides, covered with 18 × 18 mm coverslip, and frozen in liquid nitrogen for 2 min. The coverslips were removed and slides were air-dried for 10 min and permeabilized overnight in 70% ethanol at 4 °C.

For young leaves and pollen grains, samples were embedded in PEG wax and sectioned according to Wu et al. [39] with modifications. The samples were incubated with anti-H3K4me3 (AS163190, Agrisera) diluted at 1:200 in PBS containing 5% bovine serum albumin and 0.3% Triton X-100 overnight at 22 °C. The slides were washed 2 × 10 min in PBS in Coplin jars and incubated with Goat@Rabbit Alexa Fluor488(A32731R) diluted at 1:500 overnight at 4 °C. After three brief washes in PBS, the slides were subjected to a 50-90%-96% ethanol series, stained with 0.5 µg/mL 4’,6-diamidino-2-phenylindole (DAPI) in PBS with 50% glycerol, and examined on a confocal microscope.

### Chromatin immunoprecipitation

ChIP experiments were performed as described by Luo and Lam [40]. For the leaf ChIP assay, approximately 2 g of fresh leaf tissues were homogenized to a fine powder in liquid nitrogen and cross-linked with 1% formaldehyde in a nuclear isolation buffer for 10 min at room temperature, followed by the addition of 0.125 M glycine for 5 min to quench the cross-linking reaction. Next, nuclei were isolated and re-suspended in lysis buffer (50 mM Tris-HCl, 10 mM EDTA, 1% SDS, 0.1 mM PMSF, 1× protease inhibitor cocktail, pH 8.0). For MS, VN, GN, and SN ChIP assay, ~ 30 million MS, VN, SN, and 10 million GN were cross-linked with 1% formaldehyde in 1×phosphate-buffered saline (PBS) for 10 min at room temperature with gentle rotation. After quenching with glycine for 5 min, pellets of each sample were washed twice with ice-cold PBS and then resuspended in lysis buffer. The chromatin was sonicated at 4 °C with a Diagenode Bioruptor set at high intensity for 5 rounds of 10 min (30 s on, 30 s off intervals) to shear DNA approximately 100–500 bp fragments. The supernatant was first pre-cleared with mouse/rabbit IgG and then with dynabeads Protein A/G (1:1, Life technologies) for at least 1 h at 4 °C with gentle agitation. The resultant supernatant was subjected to immunoprecipitation with anti-H3K4me3 (17–614, Millipore) antibody with rotating at 4 °C overnight. The next day, the samples were incubated with dynabeads Protein A/G at 4 °C for 1 h, then laid on a magnet to collect the beads. After washing beads with a series of buffers, the immune complexes were eluted from the beads with fresh elution buffer (10 mM EDTA, 0.1 M NaHCO_3_, 1% SDS). The elutes were de-cross-linked before digestion with proteinase K. The co-immunoprecipitated materials were further purified by phenol/chloroform/isoamyl alcohol (25:24:1) and ethanol-precipitated in the presence of glycogen.

ChIP DNAs were quantified by using a Qubit fluorometer and dsDNA HS Assay Kits (Life technologies). Approximately 2 ng of ChIP DNAs were processed for library construction with input DNA as a control by using the NEBNext Ultra II DNA Library Prep Kit for Illumina (New England Biolabs). Libraries were qualified on the Agilent Bioanalyzer 2100 system and sequenced on an Illumina Novaseq 6000 system (San Diego, CA, USA) using a 150-bp paired-end strategy. Two independent biological replicates were performed for each material.

### ChIP-Seq data processing

The raw reads were trimmed by using TrimGalore version 0.6.7 [41] and processed for quality control by FastQC version 0.11.8 (http://www.bioinformatics.babraham.ac.uk/projects/fastqc/). The cleaned reads were aligned to the reference genome of tomato (ITAG4.0) by bowtie2 version 2.3.4.3 [42] and filtered for a MAPQ > 10 with samtools version 1.7 [43], and PCR duplicates were removed by using samtools version 1.7. Only uniquely mapped reads were used for further analyses. The reproducibility between biological replicates was assessed by using Pearson’s correlation coefficient based on BAM files by deepTools version 3.5.1 utility multiBamSummary [44]. The bigwig files were generated with deepTools utility bamCoverage [44], and implemented for visualization along the tomato genome using IGV version 2.12.2 [45]. The MACS2 version 2.2.7.1 program was employed to perform peak calling with a *p*-value cutoff of 0.05 [46] and replicates were evaluated by IDR version 2.0.4.2 with a cutoff of 0.05 [47]. The gene annotation of peaks was performed by the R package ChIPseeker version 1.26.2 [48].

### Identification of differential peaks and differentially modified genes

For each pairwise comparison, we combined all peaks and counted the reads mapped to each peak by using featureCount version 2.0.1. The resultant counts were used for differential peak identification with DESeq2 version 1.30.1 [49]. A peak with adjusted *q*-value < 0.05 and log_2_(fold-change) ≥ 1 was considered a differentially modified peak (DMP). Gene promoters (0–2 kb) that overlapped with the DMPs of histone modifications were considered DMP-marked genes [50, 51].

### Circos plot and gene ontology analysis

Chromosomal plots of ChIP-seq peak density with gene density and transposon (TE) were calculated in 100 kb blocks by using Circos software [52]. To determine the genomic distribution of peaks, the genome was classified into five regions including promoter (2 kb upstream of the TSS), coding exon, intron, downstream, and intergenic regions. The average ChIP-seq files were generated by using the computeMatrix and plotHeatmap utilities of deepTools version 3.5.1 [44]. For Gene Ontology enrichment, we performed a BLAST search between tomato protein sequences and Arabidopsis protein sequences (TAIR10) with a cutoff of > 40% identity and *p*-value < 0.05 and conducted gene ontology analysis using the R package clusterProfiler version 3.18.1 [47].

## Results

### Genome-wide profiling of H3K4me3 modification in SC lineage

**Fig. 1:**
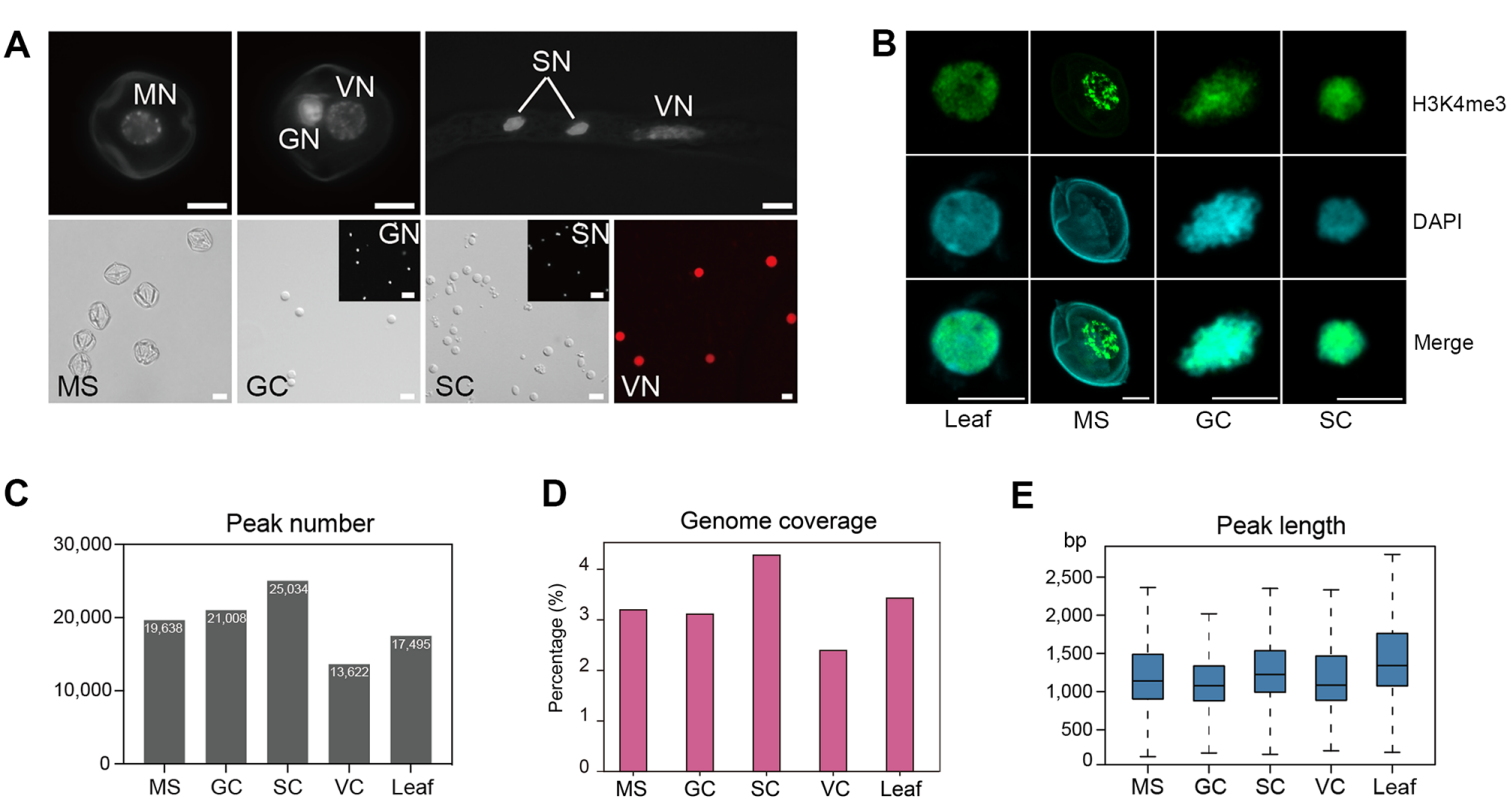
The landscape of H3K4me3 in SC lineage and somatic cells. (**A**) Top, DAPI staining of microspore, bicellular pollen, and germinated pollen. MN, microspore nuclei; GN, generative nuclei; SN, sperm nuclei; VN, vegetative nuclei. Bottom, cytological characterization of isolated tomato microspore (MS), generative cell (GC), sperm cell (SC), and VN. The VN is imaged by propidium iodide staining. Scale bar = 10 μm. (**B**) αnti-H3K4me3 and DAPI staining of cell nuclei in leaf and SC lineage. Scale bar = 5 μm. (**C**) The number of H3K4me3 peaks in MS, GC, SC, vegetative cell (VC), and leaf. (**D**) Genome coverage of H3K4me3 peaks in each sample. (**E**) The length distribution (in bp) of H3K4me3 peaks in MS, GC, SC, VC, and leaf

To assess the genome-wide profile of H3K4me3 in SC lineage cells, we conducted chromatin immunoprecipitation with sequencing (ChIP-seq) using isolated uninucleate microspores, nuclei from GCs, SCs, VC, and 4-week leaves (as representative of somatic cells) (Fig. 1A and B). On average ChIP sequencing for H3K4me3 generated 26 million reads for each biological replicate. Of which, 95.27% of clean reads were aligned to the tomato reference genome (ITAG 4.0) and 76.4% of clean reads were uniquely mapped (Table S1). The two replicates of H3K4me3 data had Pearson’s correlation coefficients > 0.92, and principal component analysis (PCA) revealed that the two replicates from each group clustered together, indicating that the ChIP-seq data were of high reproducibility (Figure S1A, S1B). To investigate genome-wide enrichment of H3K4me3 peaks in each cell type, we performed peak calling using model-based analysis of ChIP-Seq version 2 (MACS2) with a *p*-value cutoff of 0.05. There was an increase in H3K4me3 peak number in the sperm cell lineage and the SC possessed nearly twice as many H3K4me3 peaks as VC (Fig. 1C). Among all tested samples, the maximum number of H3K4me3 peaks were found in SC (25,034), covering 4.28% of the genome, while the lowest number of H3K4me3 peaks were detected in VC (13,622), covering 2.40% of the genome (Fig. 1C and D). We detected 17,495 H3K4me3 peaks in the leaf, covering about 3.43% of the genome. The H3K4me3 level in somatic cells was higher than in the VC but lower than in the SC lineage. H3K4me3 peaks tend to be narrower than other histone methylation peaks such as H3K4me1, H3K27ac, and H3K27me3 [53]. In this study, we showed that the average lengths of H3K4me3 peaks varied from 1159 bp to 1338 bp in SC lineage and VC, while the leaf H3K4me3 showed a slightly broader peak length of 1534 bp (Fig. 1E).

**Fig. 2:**
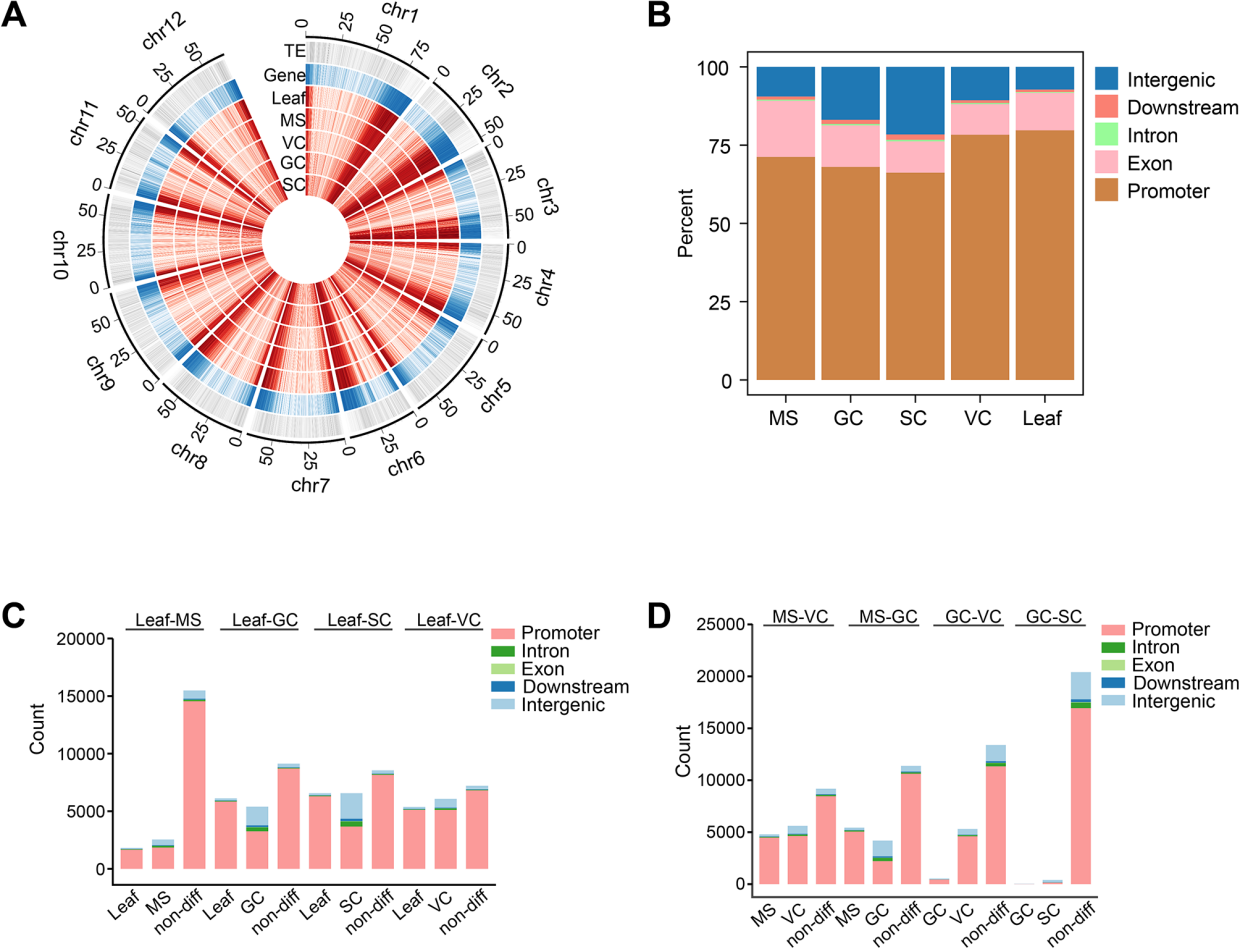
Localizations of H3K4me3 peaks during SC lineage development. (**A**) Circos-heatmap showing H3K4me3 peaks, genes, and transposable element (TE) along the whole tomato genome described in 100 kb blocks. The red tracks represent the H3K4me3 peak density, the blue track represents gene density and the grey track represents TE density. (**B**) Percentage of H3K4me3 peaks within different genomic regions in each cell type. Intergenic H3K4me3 increased in sperm cells. MS, microspore; GC, generative cell; SC, sperm cell; VC, vegetative cell. (**C, D**) Percentage of differential and non-differential H3K4me3 peaks in pairwise comparison between different cell types. The columns represent the number of enriched H3K4me3 peaks in each cell type

We used Circos to visualize the binding patterns of H3K4me3 in the genomes of each cell type. The majority of tomato genome is composed of heterochromatin and most genes are located in long contiguous stretches of gene-rich euchromatin on the distal region of each chromosome [54]. In line with this, we found H3K4me3 modifications were mainly localized in gene-rich regions of chromosome arms in leaf, MS, VC, GCs, and SCs (Fig. 2A). To determine the genomic distribution of H3K4me3 peaks in detail, we divided the genome into five regions including 2 kb promoter upstream of transcription start site (TSS), exon, intron, 2 kb downstream of transcription end site (TES), and intergenic regions. As expected, the majority (86.8%) of H3K4me3 peaks were located in genic regions, mostly in promoters (72.7%) and exons (12.6%); followed by introns, and downstream of TES which accounted for 0.4%, and 1.1% of the total peaks, respectively (Fig. 2B). On average, only 13.2% of H3K4me3 peaks were located in intergenic regions. However, the proportion of intergenic H3K4me3 peaks varied among different cell types. Notably, there was a significant (*p* < 0.01) expansion of intergenic H3K4me3 peaks as SC lineage differentiated from somatic cells. Only 7.2% of H3K4me3 peaks were located in the intergenic region in the somatic (leaf) genome, however, the percentage of intergenic H3K4me3 significantly (*p* < 0.01) increased to 9.5% in MS and continued to grow to 17.0% and 21.6% in GCs and SCs, respectively. The VC had 10.6% intergenic H3K4me3, which was comparable to that of MS.

From leaf (somatic cells) to MS, 17,207 (78.67%) of H3K4me3 peaks did not change significantly (*p* < 0.01), while the proportion of unchanged peaks is 10,782 (43.81%) in leaf vs. GC, 10,701(38.26%) in leaf vs. SC and 8,118 (39.57%) in leaf vs. VC, respectively. Although the majority of H3K4me3 dynamic changes occurred in the promoter region, 1,618 (30%) GC-enriched peaks and 2,216 (33.75%) SC-enriched peaks occurred in the intergenic regions (Fig. 2C). The comparison between VC and SC lineage showed that VC had more common H3K4me3 peaks with GC (13,387) than with MS (9,184). Between GC and VC, 90.89% (5,306) differential peaks were VC enriched (Fig. 2D).

### H3K4me3 positively correlated with transcription and negatively correlated with DNA methylation in the gene body

**Fig. 3:**
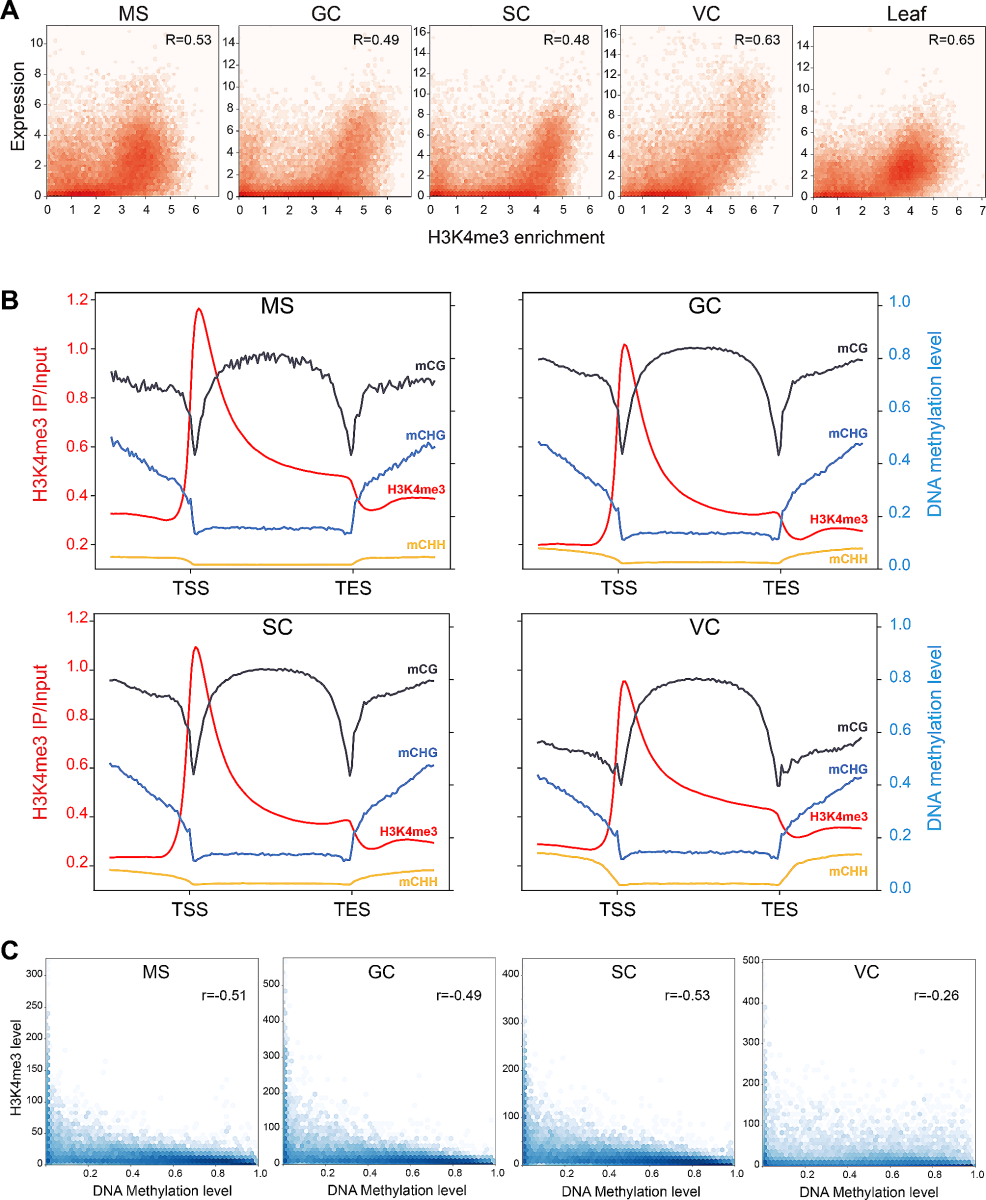
H3K4me3 is positively associated with transcription and antagonizes DNA methylation. (**A**) Scatter plots showing the relationship between the gene expression and H3K4me3 enrichment in MS, GC, SC, VC, and leaf. Pearson correlation coefficients are shown (*p*-values < 1e-10). (**B**) The distribution of H3K4me3 and DNA methylation level over genes in respective cell types (from 2 kb upstream of TSS to 2 kb downstream of TES). Average DNA methylation at CG, CHG, and CHH context on genes in SC lineage and VC are reanalyzed from data reported by Lu et al., 2021 [57]. (**C**) Correlation between H3K4me3 level and DNA methylation level at mCG context in the gene body. Pearson correlation coefficient is indicated (*p*-value = 0). MS, microspore; GC, generative cell; SC, sperm cell; VC, vegetative cell

To evaluate the active effects of H3K4me3 on gene expression in each cell type, we revisited our robust RNA-seq dataset generated from the same cell types [55]. Our re-annotation of the Heinz1706 genome predicted 53,666 protein-coding genes, of which 19,710 (36.7%) in MS, 19,431 (36.2%) in GC, 21,195 (39.5%) in SC, 14,843 (27.7%) in VC, and 18,715 (34.9%) in leaf were associated with a least one H3K4me3 peak respectively. In SC lineage, each protein-coding gene was marked with 0.91 peak, which was slightly higher than that in VC and leaf (Figure S2). Integrative analysis of H3K4me3 enrichment with RNA-seq FPKM (fragments per kilobase of transcript per million fragments mapped) revealed an overall positive correlation between H3K4me3 enrichment and gene expression levels in all examined cell types (Fig. 3A). The strength of correlation varies from sample to sample. There are relatively strong positive correlations in the VC and leaf, with a correlation coefficient of 0.63 and 0.65, respectively. In contrast, a moderate positive correlation was detected in GC and SC, with a correlation coefficient of 0.49 and 0.48, respectively. These suggest H3K4me3 enrichment is a better indicator of gene expression in somatic or vegetative cells, than in SC lineage cells.

The interplay between histone methylation and DNA methylation (in CG, CHG, CHH context; H = A, C, or T) largely decides the epigenetic regulation of gene expression. To gain insight into the potential relationship between H3K4me3 and DNA methylation, we created the average meta-gene profile by dividing the transcribed region (from TSS to TES) and 2 kb flanking region into 160 bins. The result revealed that H3K4me3 peaks were enriched immediately downstream of the transcription start sites (TSS), with a maximum at bin 42 in GCs and SCs; and at bin 43 in leaf, MS, and VC (Fig. 3B). The mCG showed a remarkable valley at bin 41 in all cell types, while mCHG and mCHH showed a noticeable decline at bin 41–42. This result was in line with previous observations on the localization of H3K4me3 in Arabidopsis seedlings [56]. Intriguingly, the deposition of H3K4me3 antagonizes DNA methylation levels near the 3’ proximal of the TSS, where both CG and non-CG methylations (mCHG, mCHH) show a sharp decline (Fig. 3B).

To explore the correlation between H3K4me3 and DNA methylation in the promoter and gene body region, the Pearson correlation coefficient was calculated using the FPKM of H3K4me3 and DNA methylation level. In the gene body, a moderate negative correlation was detected between mCG DNA methylation and H3K4me3 in MS, GC, and SC, with correlation coefficients of -0.51, -0.49, and − 0.53, respectively (Fig. 3C). A slight negative correlation was detected between the mCG context and H3K4me3 in VC. In contrast, no significant correlations (**|**r**|** > 0.2, *p* < 0.01) were observed in mCHH and mCHG contexts. In the promoter, no significant correlation was observed between all three DNA methylation contexts and H3K4me3 level (Table S2). These results suggest a mutual exclusion relationship between H3K4me3 and DNA methylation occurred only in the mCG context of the gene body.

### Regulation of H3K4me3 deposition following asymmetric division of microspore

**Fig. 4:**
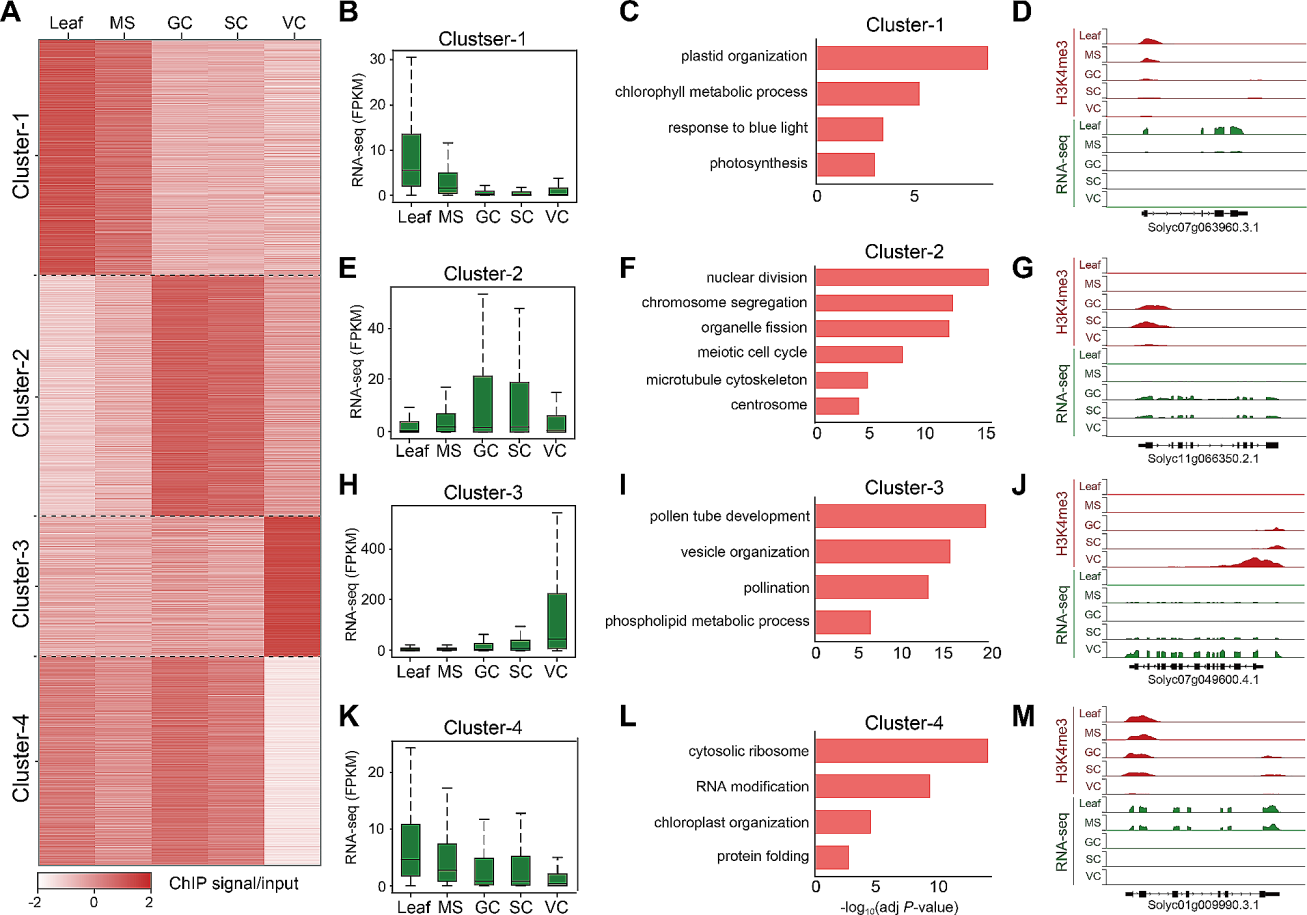
Clustering of H3K4me3 associated genes in each sample. (**A**) Heat maps showing the dynamics of H3K4me3 domains in SC lineage and somatic tissue. The colors represent the log_2_-transformed H3K4me3 ratio scaled by row. (**B, E, H, K**) Box plot for the expression level of gene clusters 1 to 4. The center of the box plots represents the median value and the lower and upper lines represent the 5% and 95% quantiles, respectively. (**C, F, I, L**) Gene ontology analysis of genes in clusters 1 to 4. (**D, G, J, M**) The genome browser view of the log_2_-transformed H3K4me3 enrichment ratio and the normalized RNA-seq read counts on representative genes. MS, microspore; GC, generative cell; SC, sperm cell; VC, vegetative cell

The asymmetric cell division of the microspore is determinative for SC lineage development and gives rise to GCs and VC with distinct identities and fates. To study H3K4me3-associated transcription during SC lineage development, we categorized H3K4me3-associated genes into four clusters by using the H3K4me3 signal strength around the 2 kb flanking region of TSS (Fig. 4A, Table S3). The result showed microspores still retain somatic H3K4me3 profiling, while GCs and SCs share similar H3K4me3 features in terms of protein-coding gene expression. Genes in cluster 1 showed high expression levels in leaf and MS (Fig. 4B) with significantly enriched gene ontology (GO) terms for plastid organization, chlorophyll metabolic process, response to blue light, and photosynthesis (Fig. 4C). For instance, the genome browser view showed significant H3K4me3 enrichment at the first exon of Solyc07g063960.3.1 (encoding a ribosomal protein L24) in the leaf and MS, but not in the SC lineage and VC (Fig. 4D). Genes in cluster 2 showed high levels of H3K4me3 in GCs and SCs (Fig. 4E) including genes significantly enriched for nuclear division, chromosome segregation, organelle fission, mitotic cell cycle, microtubule cytoskeleton, and centrosome-related GO terms (Fig. 4F) [i.e. Solyc11g066350.2.1, SHUGOSHIN 2, a conserved protein to protect sister chromatid cohesion during mitosis and meiosis (Fig. 4G)]. Moreover, MYB transcription factor DUO1 POLLEN1 (DUO1), a master regulator of cell cycle progression of GCs [58] and DUO1-activated target genes *GCS1*, *GEX2*, *TIP5;1* [59] also showed synchronized upregulation in H3K4me3 deposition and gene expression, suggesting an H3K4me3 activated regulation of DUO1-module following microspore asymmetrical division.

Gene cluster 3 was preferentially expressed in VC (Fig. 4H), including genes for pollen tube development, vesicle organization, pollination, and phospholipid metabolic process (Fig. 4I) [i.e. Solyc07g049600.4.1, FIMBRIN-5, an actin-bundling factor required for the organization of actin cytoskeleton (Fig. 4J)]. Genes in cluster 4 appeared to be sparsely expressed in VC but constitutively expressed in other SC lineage cells and somatic cells (Fig. 4K). These genes had enriched terms for the ribosome, RNA modification, protein folding, and chloroplast organization (Fig. 4L) [i.e. Solyc01g009990.3.1., Peptidyl-prolyl cis-trans isomerase, an enzyme catalyzes the cis-trans isomerization of the prolyl peptide bond. (Fig. 4M)] Together, these results indicated a significant difference in H3K4me3 patterns between the microspore and its daughter cells. Asymmetric division of microspore transforms the H3K4me3 from somatic to reproductive profiling.

Analysis of unchanged H3K4me3 peaks across different cell types suggested that genes related to ‘cellular response to DNA damage stimulus’, ‘DNA repair’, and ‘RNA splicing’ maintained constitutive expression in somatic cells and SC lineage development (Figure S3). From somatic cells to VC, H3K4me3 remained unchanged in genes related to ‘mitochondrion organization’ and ‘helicase activity’ (Figure S3A). From MS to SC, H3K4me3 remained unchanged in genes related to ‘mRNA splicing, via splicesome’ and ‘Golgi vesicle transport’ (Figure S3B).

### Selective loss of H3K4me3 in genes related to cell cycle and hormone signaling contributes to gene regulation

**Fig. 5:**
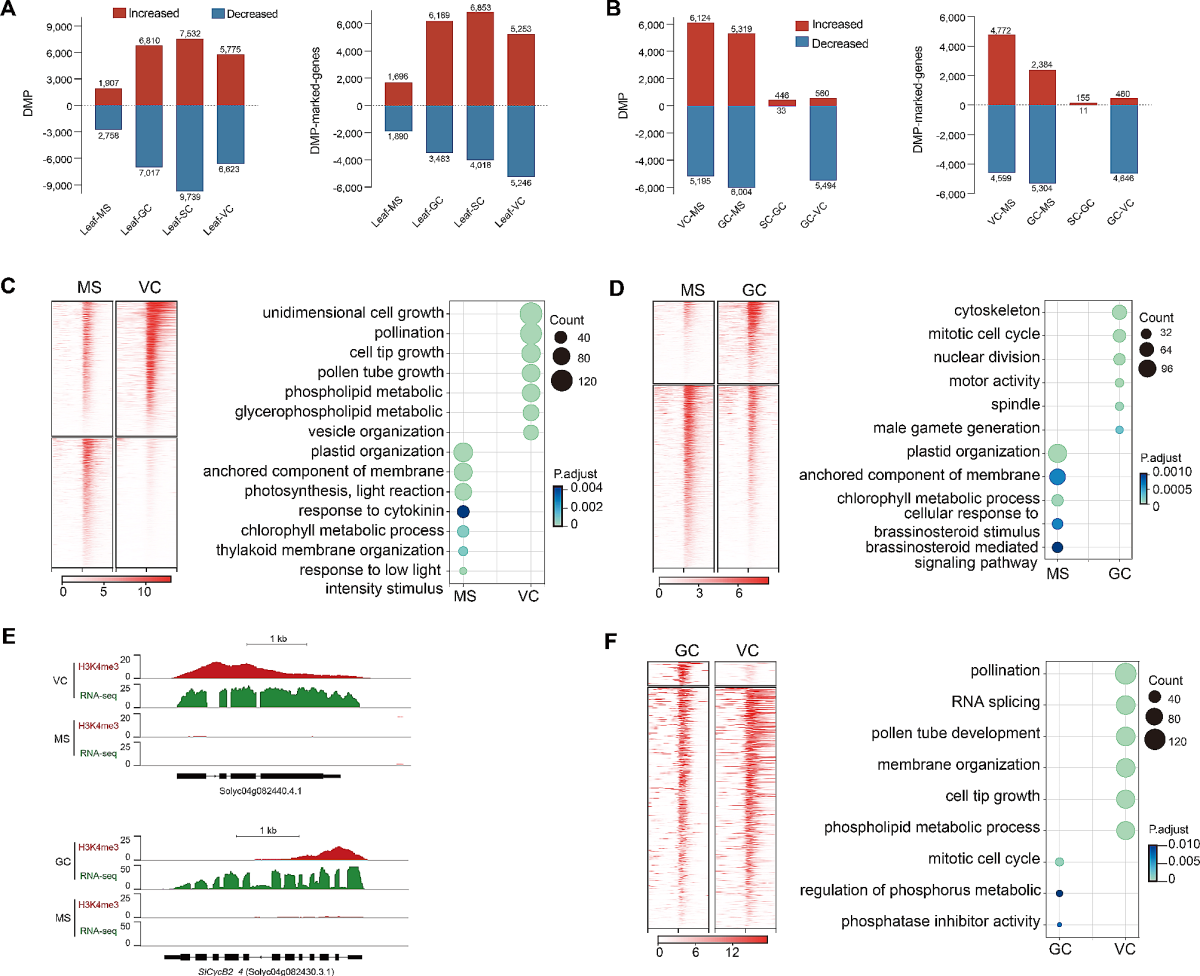
Pairwise comparison of H3K4me3 in different cell types. (**A, B**) Graphs showing the number of differentially modified H3K4me3 peaks (DMPs) and DMP-marked genes in the comparisons of MS versus VC, MS versus GC, and GC versus VC. (**C, D, F**) Enriched GO terms of genes with increased H3K4me3 signals in the MS versus VC (**C**), MS versus GC (**D**), and GC versus VC (**F**). (**E**) The genome browser view of H3K4me3 signals and gene expression near Solyc04g082440.4.1 in the MS and VC; and *SlCycB2_4* (Solyc04g082430.3.1) in the MS and GC

To further investigate the effects of H3K4me3 deposition in different cell types, we detected differentially modified peaks (DMPs, adjusted q-value < 0.05 and log_2_Foldchange ≥ 1) and genes marked by these DMPs (gene promoter region overlapped with at least one DMP) in a pairwise manner (Table S4 and S5). Consistent with the correlation matrix and gene clustering in which the H3K4me3 pattern of somatic cells is more similar to that of MS than GCs, SCs, and VC. A small number of DMPs and DMP marked genes were identified in the leaf versus MS, while approximately 2 ~ 4 times of DMPs and DMP marked genes were identified in the leaf versus GCs, SCs, or VC (Fig. 5A). Notably, there are 7,017 and 9,739 decreased DMPs were detected in the leaf, compared to GCs and SCs, respectively. However, only 3,483 and 4,018 decreased DMP-marked genes were detected in the leaf, compared to GCs and SCs. This is mainly caused by an increased proportion of intergenic DMPs in GCs and SCs (Fig. 2C).

Compared with the MS, VC had 6,124 up-regulated H3K4me3 DMPs which marked 4,772 genes, and 5,195 down-regulated DMPs which marked 4,599 genes; the GCs had 5,319 up-DMPs, which marked 2,384 genes, as well as 6,004 down-DMPs, which marked 5,304 genes (Fig. 5B). This suggests extensive gene activation and repression occurred in the two daughter cells of MS. Intriguingly, in the GCs versus the VC, over 90% of DMPs (5,494) and DMP-marked genes (4,646) showed downregulation in GCs, suggesting a possible repression of GC-specific genes by selectively losing of H3K4me3. There were only 479 DMPs and 166 DMP-marked genes were detected in SCs versus GCs, suggesting the H3K4me3 landscape in SCs was probably inherited from GCs.

Up-DMPs-marked genes in the MS versus VCs were mainly enriched for photosynthesis (in MS) and pollen tube growth (in VC) related terms, including Solyc04g082440 (U-box domain-containing protein) which was marked by H3K4me3 across the whole coding region (Fig. 5C, E). Notably in MS, the GO term ‘response to cytokinin’ was highly represented (Table S6), including genes encoding histidine kinases (Solyc07g047770, Solyc05g015610, Solyc04g008110), histidine-containing phosphotransfer protein 1(Solyc01g098400.3, Solyc06g084410.4, Solyc01g080540.3), two-component response regulator ORR (Solyc11g071630, Solyc05g014260) and ARR (Solyc05g054390, Solyc10g079600, Solyc04g008050, Solyc06g048930). This suggests selective loss of H3K4me3 from cytokinin signaling loci may contribute to the VC gene regulation.

Up-DMPs-marked genes in the MS versus GCs had enriched terms associated with mitotic cell cycle and male gamete generation (in GCs), including cyclin-encoding gene *SlCycB2_4* (Solyc04g082430) which is involved in the regulation of mitotic activity (Fig. 5D, E); Among five classical plant hormones, only brassinosteroid (BR) mediated signaling pathway was overrepresented in MS (Table S7), including BR receptor *BRI1* (Solyc04g051510.1), *BES1/BZR1* (Solyc02g063010.3, Solyc03g005990.3), *BSK1* (Solyc04g082260.4), *BIN2* (Solyc03g006070.3); and BR regulated receptor-like kinase *THESEUS 1* (Solyc10g006870.1, Solyc05g054680.1), *HERK 1* (Solyc06g005230.3, Solyc05g054860.1) which are required for cell elongation during vegetative growth. This suggests selective loss of H3K4me3 from BR signaling loci may contribute to the gene regulation in GCs.

Furthermore, Up-DMPs-marked genes in GCs versus VC were enriched for mitotic cell cycle, phosphatase inhibitor activity, and regulation of phosphorus metabolic process (GC), while Up-DMPs-marked genes in the VC versus GCs had enriched terms associated with pollination, RNA splicing, pollen tube development, membrane organization, cell tip growth, phospholipid signaling, and pollination (Fig. 5F). In summary, these results revealed that H3K4me3 deposition and eviction are correlated with the functional characteristics of different cell types. H3K4me3-associated gene expression significantly changed after asymmetric division, and largely settled in the GCs.

## Discussion

Accumulating genetic and omics studies have revealed that epigenetic regulations play a central role in the cell fate transition from somatic to reproductive cells [7–9, 12, 32], but less is known about the H3K4me3 dynamics during SC lineage development in higher plants, partly because the purification of generative cells from tri-cellular pollen species (i.e. Arabidopsis, rice, and maize) is technically challenging. In this work, we successfully purified MS, GC, and SC covering three sequential differentiation stages of SC lineage, and used ChIP sequencing to characterize a comprehensive map of the H3K4me3.

H3K4me3 distribution in the genome of *Arabidopsis thaliana* and tomato is largely conserved. In both species and different cell types in tomato, H3K4me3 is predominantly enriched in genic regions, especially around transcription start sites, and generally associated with active transcription. However, about 60% of the tomato genome consists of repetitive sequences (mostly transposable elements) [38], whereas the *Arabidopsis thaliana* genome has ~ 24% repetitive sequences [60]. The difference in repeat content caused variations in the H3K4me3 profile between tomato and Arabidopsis. For instance, the H3K4me3 peak covers 2.4%~4.28% of the tomato genome, which is lower than the 12.1% coverage in Arabidopsis. 86.8% vs. 95.7% of H3K4me3 peaks were located in genic regions in tomato and Arabidopsis, respectively. In tomato, 18,715 ~ 21,195 genes are associated with the H3K4me3 peak, while a comparable 14,712 genes with H3K4me3 peaks in Arabidopsis. The expansion of intergenic H3K4me3 peaks along with SC lineage development is a distinct feature from somatic cells. In rice (*Oryza sativa* L. japonica cv Nipponbare) seedlings, only 14.7% of H3K4me3 was located in the intergenic region [61].

We previously demonstrated that tomato GCs and SCs have almost identical DNA methylomes [57]. Consistently, here we showed that GCs and SCs shared a similar H3K4me3 deposition at protein-coding genes, suggesting both DNA methylation and H3K4me3 in SCs were probably inherited from GC. Consistent with the report in rice [61], we found H3K4me3 modifications were significantly enriched at 3’ proximal of TSS but seldom near TES. Through an integration analysis of histone modification and DNA methylation datasheets, we found that H3K4me3 negatively correlated with DNA methylation at 3’ proximal of TSS. It has been reported in animals that during gametogenesis H3K4 methylation and DNA methylation are mutually exclusive and removal of H3K4 methylation is required for proper DNA methylation establishment at imprinted CpG islands because H3K4 strongly inhibited the interaction between DNA methylation regulators and the extreme amino terminus of histone H3 [62, 63]. Interestingly, unlike CG methylation, non-CG methylation did not show a significant mutual exclusion with H3K4me3. This may be explained by non-CG methylation mostly occurring in intergenic regions, whereas a low level of non-CG methylation, in particular mCHH was less abundant in the gene body.

Our results supported that H3K4me3 in general positively correlated with gene expression. Compared to GCs and SCs, VC featured rapid tip growth and active cellular metabolism but surprisingly showed the least H3K4me3 enrichment. This suggests active cellular metabolism does not necessarily reflect active gene expression, and other epigenetic regulations, for instance, H1 eviction may also impact the gene expression in VC. Many evidence lines have shown that the epigenetic differences in the nuclei of the pollen cells contribute to their diverse functions. The VN with diffused chromatin is proposed to benefit transposable element (TE) expression which may generate small interfering RNAs (siRNA) to inhibit TEs activities in the SC [64]. Whereas, the GN and SN show condensed chromatin which is suggested to be a disadvantage of gene expression. We detected fewer H3K4me3 peaks and genome coverage in VC, but how the loss of H3K4me3 related to TE reactivation in VC still needs exploration.

GO ontology of differential H3K4me3 marked genes during MS-GC transition showed that brassinosteroid (BR)-mediated signaling was repressed in GC. BRs are steroid-like plant hormones essential for plant reproductive development by regulating anther formation and pollen germination. In Arabidopsis, evidence indicates that BR production plays an important role in the initiation of female gametogenesis [65], and BR signaling is required for finetune female germline specification. Inactivation of BR signaling in somatic cells or activation of BR signaling in MMCs led to multiple MMC formations [66]. However, little is known about the role of BR during male germline specification. Consistent with its role in the sporophyte-to-gametophyte transition, our ChIP-seq data suggest BR production and signaling may also play a key role in SC lineage specification. Intriguingly, THESEUS 1 and HERK1 belong to the CrRLK family which serve as cell-wall integrity sensors [67]. The loss of H3K4me3 on genes involved in the BR pathway such as *BES1/BZR1*, *THESEUS 1*, and *HERK1* might inhibit cell growth and lead to transcriptional repression during the maturation of sperm cells.

H3K4me3 is associated with gene activation and we have shown using immunoblot that SC has a lower level of H3K4me3 compared to GC [55]. In this study, instead of a lower level of H3K4me3 deposition, we detected a higher H3K4me3 peak number and genome coverage in SC, which is puzzling. Here, nuclear protein rather than total protein (including cytoplasmic protein) is used for immunodetection of H3K4me3 in GCs and SCs. The higher number of H3K4me3 peaks in SC may be preparing sperm genes for zygotic expression. It is also possible that pre-configuration of sperm genes with H3K4me3 might benefit the rapid transcription of paternal genes after double fertilization [68].

**Fig. 6:**
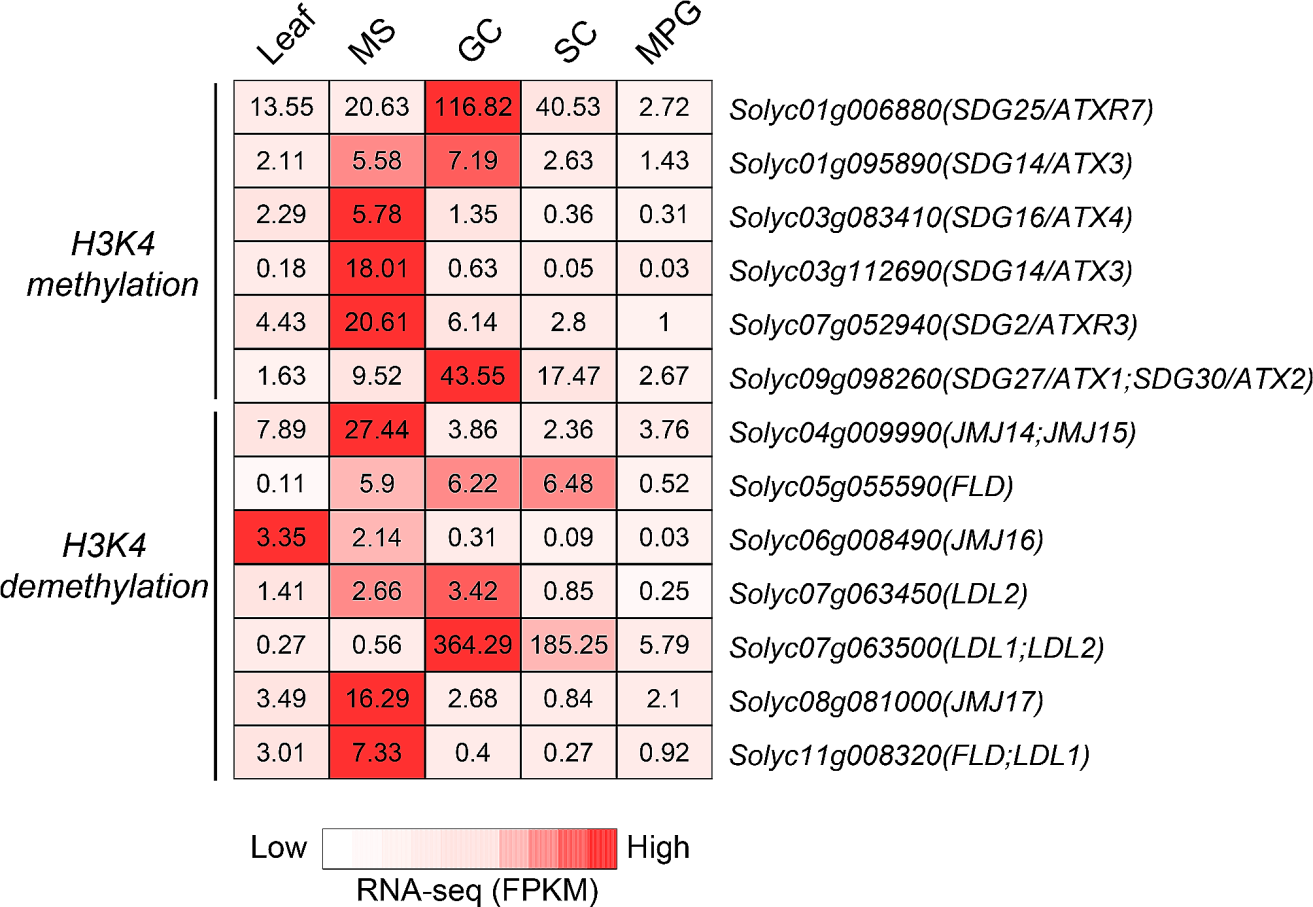
Expression of putative H3K4 regulators during pollen development. The mean expression level (fragments per kilobase of transcript per million fragments mapped, FPKM) for tomato homologs of putative histone H3K4 methylases and demethylases are indicated in each cell. RNA-seq data are from Liu et al. 2018 [4]. MS, microspore; GC, generative cell; SC, sperm cell; MPG, mature pollen grain

In Arabidopsis, class III of SET domain group (SDG) are specific H3K4 methyltransferases, including *SDG27*, *SDG30, SDG14*, *SDG16*, *SDG29*, *SDG2*, and *SDG25*. Among them, tomato homologs of *SDG25/ATXR7* (Solyc01g006880) and *SDG27/ATX1* (Solyc09g098260) show preferential expression in GC and SC (Fig. 6). We also detected seven putative H3K4 demethylases, homologous to Arabidopsis *FLD*, *LDL1*, *LDL2*, and *JMJ14 to JMJ18* [25]. One putative H3K4 demethylase (Solyc07g063500.4.1) homogenous to *LDL1* and *LDL2* is preferentially expressed in GC and SC. This observation suggests that histone methylation and demethylation on H3K4 are highly dynamic in the development of SC lineage.

We previously revealed that the microspore is more similar in gene expression profiles to somatic cells than its specialized daughter GCs or SCs, and GCs are nearly identical to SCs [4]. In this study, we further confirmed this conclusion at the epigenetic level that the microspore and leaf also have parallel H3K4me3 patterns with somatic cells, and H3K4me3 in SCs is largely settled at the GC stage. Our finding will help investigate the molecular mechanisms underlying SC lineage development in plant species and provide new resources for cell identity establishment and differentiation.

## Conclusion

Here we provided an H3K4me3 landscape covering the whole development stages of SC lineage. We observed an enrichment of H3K4me3 peaks at 3’ proximal of TSS, which is independent of cell types. H3K4me3 is positively associated with transcript abundance, however, the H3K4me3 level is more reliable in predicting gene expression in somatic cells and VC, than SC lineage. H3K4me3 is mutually exclusive with DNA methylation at 3’ proximal of TSS. Asymmetrical division is a decisive event in the genome-wide relocation of H3K4me3. Microspores are similar to somatic cells in the H3K4me3 profile, whereas sperm cells share nearly identical H3K4me3 with generative cells. Gene ontology analysis revealed selective loss of H3K4me3 in genes related to cell cycle and hormone signaling may play an important role in SC lineage differentiation.

### Electronic supplementary material

Below is the link to the electronic supplementary material.

Supplementary Material 1

Supplementary Material 2

Supplementary Material 3

Supplementary Material 4

Supplementary Material 5

Supplementary Material 6

Supplementary Material 7

Supplementary Material 8

## Data Availability

ChIP-seq data are available in the Genome Sequence Archive (https://bigd.big.ac.cn/gsa/) under the accession number CRA015675 or via Shared URL: https://ngdc.cncb.ac.cn/gsa/s/O372MI3A.

## Abbreviations

ChIP-seq: Chromatin immunoprecipitation sequencing
H3K4me3: Trimethylation of histone H3 lysine 4
MS: Microspore
GC: Generative cell
SC: Sperm cell
VC: Vegetative cell
TSS: Transcription start site
TES: Transcription end site
DMP: Differentially modified peaks
FPKM: fragments per kilobase of transcript per million fragments mapped
