# Supplementary material for: Whole-genome landscape of histone H3K4me3 modification during sperm cell lineage development in tomato
Source: BMC Plant Biol. 2024 Jun 27;24:610. doi: 10.1186/s12870-024-05318-8 (PMC11210149; doi:10.1186/s12870-024-05318-8)
Supplement: Supplementary file 8 — Supplementary Material 8 [file 12870_2024_5318_MOESM8_ESM.docx]

**Supporting information**


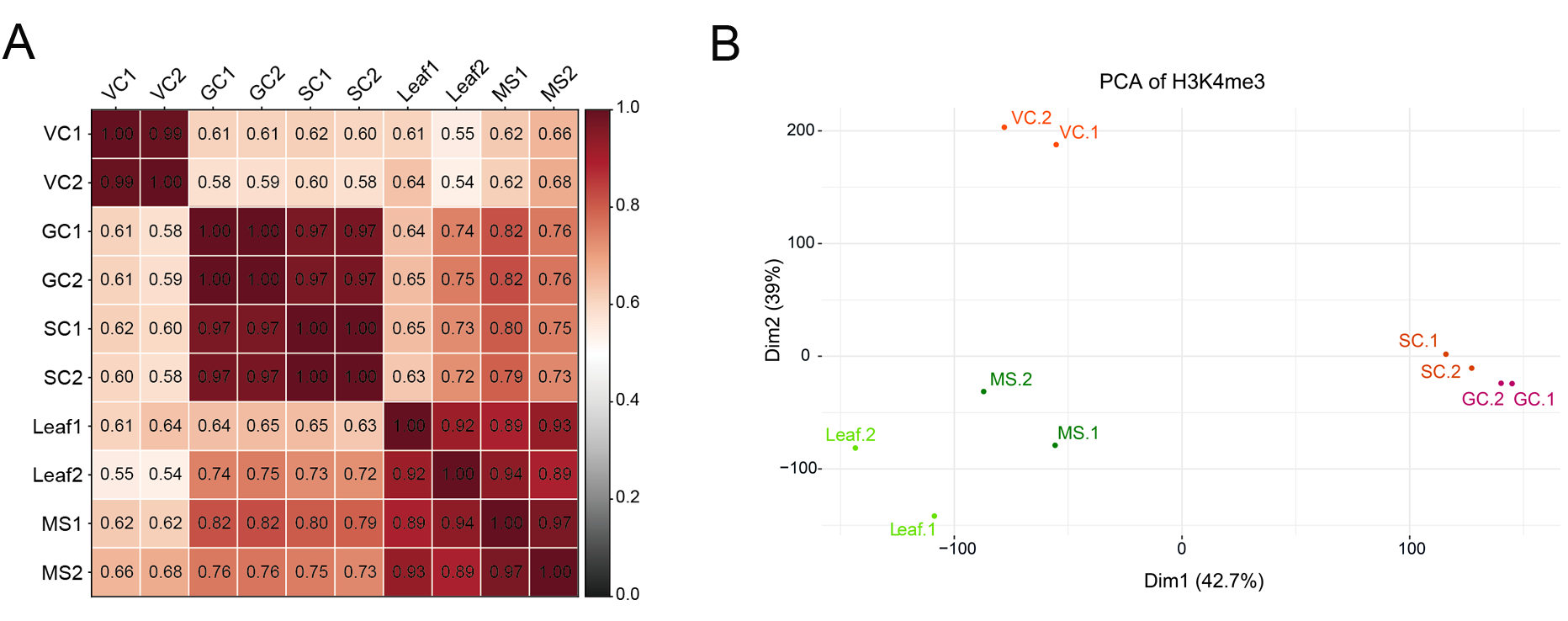


**Figure S1**. Reproducibility of the H3K4me3 ChIP-seq datasets.

(A) Pearson cross-correlation matrix of the ChIP-seq datasets. The number represents the Pearson correlation coefficient, each with two replicates. (B) Principal component analysis of ChIP-seq datasets in indicated samples, each with two biological replicates


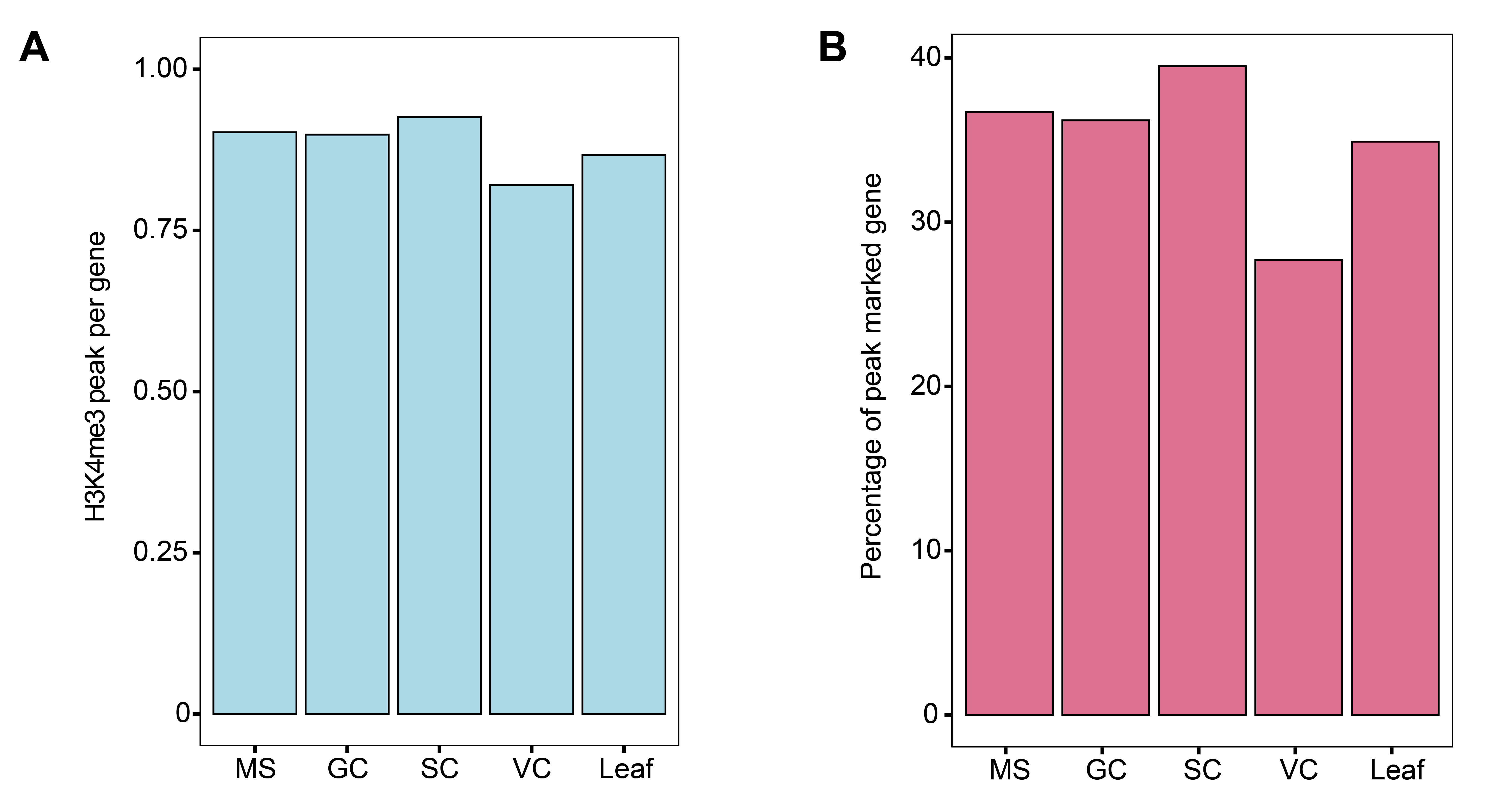


**Figure S2.** Statistics of H3K4me3 peaks on protein-coding genes.

(A) H3K4me3 peak per gene in different cell types. The number of H3K4me3 peaks that fall in genic regions (promoter, exon, intron, downstream) is divided by the number of genes overlapped with at least one H3K4me3 peak. (B) Percentage of H3K4me3 marked genes in all 53,666 protein-coding genes.


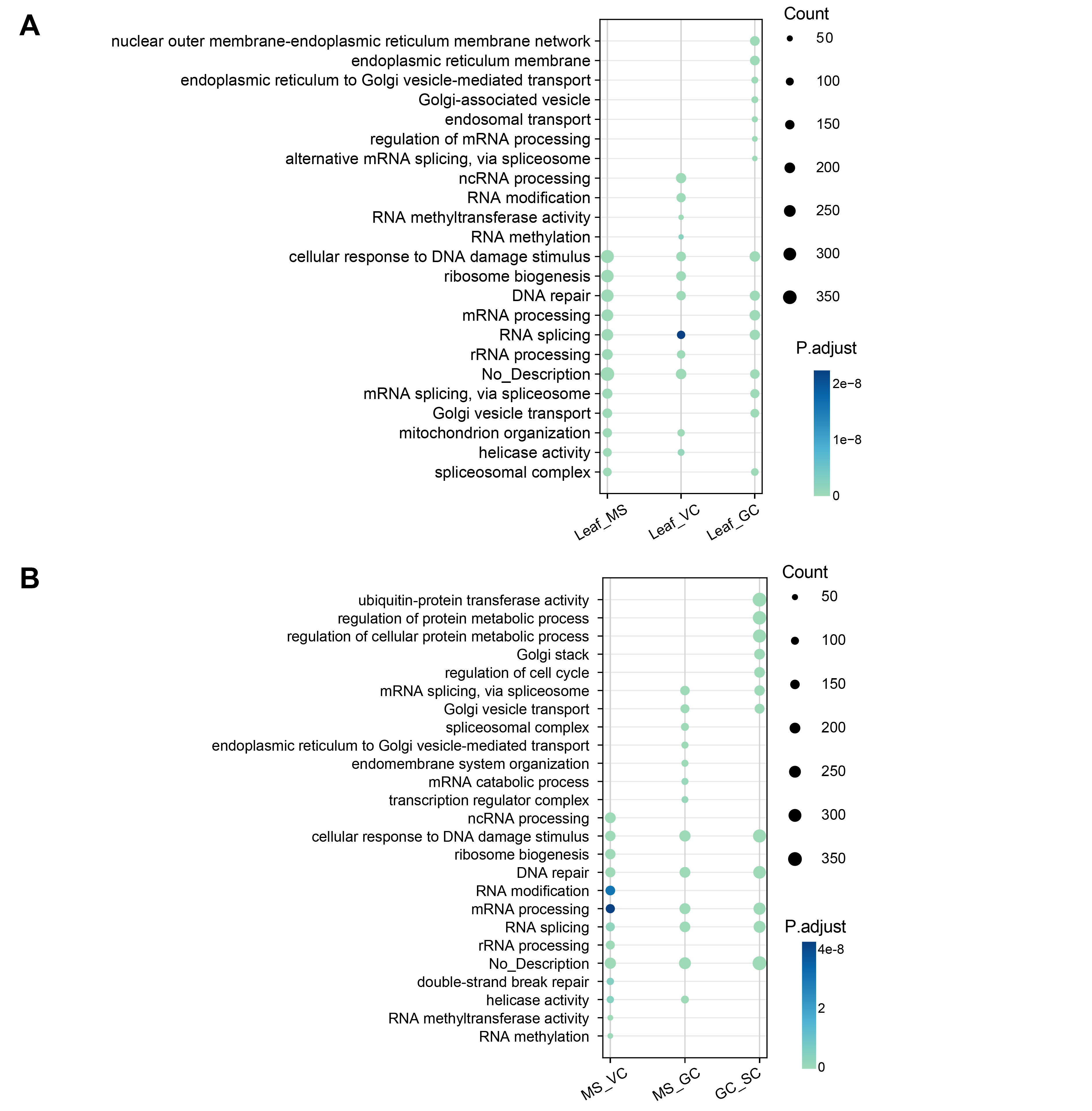


**Figure S3.** Gene ontology of genes with non-differential H3K4me3 peaks in pairwise comparison.
